# Supplementary material for: Binocular treatment for amblyopia: A meta-analysis of randomized clinical trials
Source: PLoS One. 2021 Oct 8;16(10):e0257999. doi: 10.1371/journal.pone.0257999 (PMC8500435; doi:10.1371/journal.pone.0257999)
Supplement: Attachment — Submitted filename: Response to Reviewers R2.docx [file pone.0257999.s003.docx]

**Response to Reviewers**

We appreciate the Editorial Board Member and the Reviewers for the opportunity to revise our work for consideration for publication on PlosOne.

This is the reply to Reviewer #2:

1. **A paragraph should be added to the discussion comparing this study to other recently published meta analyses in the field. This will help to place the study in the context of the current literature.**

We added in the discussion paragraph.

2. **Newly added text in the abstract and discussion should be proof read for grammar and word choice.**

Ok, we did.
